# Relationship between heart failure and intestinal inflammation in infants with congenital heart disease

**DOI:** 10.1186/s12866-024-03229-0

**Published:** 2024-03-25

**Authors:** Qi-Liang Zhang, Xiu-Hua Chen, Si-Jia Zhou, Yu-Qing Lei, Qiang Chen, Hua Cao

**Affiliations:** 1 Department of Cardiac Surgery, Fujian Maternity and Child Health Hospital College of Clinical Medicine for Obstetrics & Gynecology and Pediatrics, Fujian Medical University, Fuzhou, China; 2 Fujian Children’s Hospital (Fujian Branch of Shanghai Children’s Medical Center), College of Clinical Medicine for Obstetrics & Gynecology and Pediatrics, Fujian Medical University, Fuzhou, China

**Keywords:** Congenital heart disease, Heart failure, Intestinal microbiota, Intestinal inflammation, Infant

## Abstract

**Objective:**

The association between heart failure (HF) and intestinal inflammation caused by a disturbed intestinal microbiota in infants with congenital heart disease (CHD) was investigated.

**Methods:**

Twenty infants with HF and CHD who were admitted to our hospital between October 2021 and March 2022 were included in this study. Twenty age- and sex-matched infants without HF at our hospital were selected as the control group. Faecal samples were obtained from each participant and analysed by enzyme-linked immunoassay and 16 S rDNA sequencing to assess intestinal inflammatory factors and the microbiota.

**Results:**

The levels of intestinal inflammatory factors, including IL-1β, IL-4, IL-6, IL-17 A and TNF-α, were greatly increased, while the levels of IL-10 were significantly decreased in the HF group compared to the control group (*p* < 0.05). The intestinal microbial diversity of patients in the HF group was markedly lower than that in the control group (*p* < 0.05). The abundance of *Enterococcus* was significantly increased in the HF group compared to the control group (*p* < 0.05), but the abundance of *Bifidobacterium* was significantly decreased in the HF group compared to the control group (*p* < 0.05). The diversity of the intestinal microbiota was negatively correlated with the levels of IL-1β, IL-4, IL-6 and TNF-α in the intestinal tract but was positively correlated with that of IL-10. The abundance of *Enterococcus* was positively associated with the levels of IL-1β, IL-4, IL-6 and TNF-α in the intestinal tract but was negatively correlated with that of IL-10. NT-proBNP was positively associated with the levels of IL-1β, IL-4, IL-6 and TNF-α in the HF group but was negatively correlated with that of IL-10. The heart function score was positively associated with the levels of IL-1β, IL-4, IL-6 and TNF-α in the HF group but was negatively correlated with that of IL-10.

**Conclusions:**

Infants with CHD-related HF had a disordered intestinal microbiota, decreased diversity of intestinal microbes, increased levels of pathogenic bacteria and decreased levels of beneficial bacteria. The increased abundance of *Enterococcus* and the significant decrease in the diversity of the intestinal microbiota may exacerbate the intestinal inflammatory response, which may be associated with the progression of HF.

## Introduction

Heart failure (HF) is a serious disease [1]. Because the pathophysiological mechanism of HF is very complex and includes haemodynamic abnormalities, cardiac remodelling, inflammation, and activation of the neuroendocrine system [2], the mechanism of HF is not clear [3, 4]. With further research on HF, the pathogenesis of HF has changed from a “neurohumoural mechanism” to “neurohumoural, immunological and multitarget regulatory mechanisms” [5]. Inflammation is an important factor that affects the pathogenesis of HF and is an important target for the treatment of HF [6, 7]. HF is considered a systemic inflammatory disease characterized by significantly increased proinflammatory factor levels and reduced anti-inflammatory factor levels [8, 9]. The gut is an important source of these inflammatory factors.

The intestinal tract is composed of many microorganisms and is the largest immune organ and endocrine system in the human body [10, 11]. HF caused by the redistribution of blood can cause oedema in the intestinal wall and intestinal epithelial ischaemic damage, which leads to a disturbed intestinal microbiota and increased intestinal permeability. Therefore, additional inflammatory factors secreted by intestinal pathogenic bacteria enter the blood circulation, aggravating the systemic inflammatory response and exacerbating the progression of HF.

Congenital heart disease (CHD) is the most common cause of HF in children [12]. HF in patients with severe CHD often occurs in infancy. To date, no study has examined the effect of intestinal inflammation on the progression of HF in infants with CHD. Therefore, we aimed to examine the association between HF and intestinal inflammation in infants with CHD. We hypothesized that HF in infants with CHD could lead to a disordered intestinal microbiota, which could aggravate the intestinal inflammatory response. Moreover, intestinal inflammatory factors entering the blood circulation aggravate the systemic inflammatory response and accelerate the progression of HF.

## Methods

### Research design

This study was designed to examine the association between HF and intestinal inflammation caused by a disturbance in the intestinal microbiota in infants with CHD. Left-to-right shunt CHD is the most common type of CHD, accounting for approximately 50 to 70% of CHD cases. Severe HF often leads to congestive HF in infants. Infants with left-to-right shunt CHD and congestive HF were selected as the study subjects. This study was the first to examine the relationship between HF and intestinal inflammation in infants with CHD. Since no similar study has been performed, it was difficult to find adequate data in the literature for sample size calculation. Being an exploratory study, here the sample size could not be calculated. Twenty infants with HF and CHD who were admitted to our hospital between October 2021 and March 2022 were included in this study. Twenty age- and sex-matched infants without HF were selected as the control group. We used the modified Ross score and NT-BNP to evaluate the severity of HF.

The inclusion criterion was HF caused by left-to-right shunt CHD. The exclusion criteria were as follows: (1) patients with other serious diseases, such as digestive tract malformation, kidney failure, or liver failure; (2) patients with digestive tract diseases, such as diarrhoea, constipation, or jaundice; (3) patients with infection or who were using antibiotics; and (4) patients whose parents refused to participate in this study.

### Faecal sample collection

Fresh faecal samples were obtained using a faecal collector and were transported in a liquid nitrogen tank. The faecal samples were stored in at − 80 ℃.

### Experimental methods

#### 16 S rDNA sequencing

Total genomic DNA samples were prepared with the M5635-02 OMEGA Soil DNA Kit (Omega Bio-Tek, Norcross, GA). The extracted DNA was selected as a template. PCR amplification of bacterial 16 S rRNA genes (V3–V4 region) was performed, and a PCR amplification library was subsequently constructed. A NovaSeq 6000 SP Reagent Kit (500 cycles) from Suzhou PANOMIX Biomedical Tech Co., Ltd., was used for sequencing on the Illumina NovaSeq platform. The sequence data were analysed by using the QIIME2 and R packages (v3.2.0).

#### Enzyme-linked immunosorbent assay (ELISA) analysis of intestinal inflammatory factors

Intestinal inflammatory factor levels were measured with ELISA kits. Human interferon IL-1β, IL-4, IL-5, IL-6, IL-8, IL-10, IL-12, IL-17 A, IFN-α, IFN-γ, and TNF-α ELISA kits were used to determine the levels of IL-1β, IL-4, IL-5, IL-6, IL-8, IL-10, IL-12, IL-17 A, IFN-α, IFN-γ and TNF-α, respectively, in the faecal samples.

The faecal samples were maintained at 2–8 °C after being thawed. PBS (pH 7.4) was added, and the samples were homogenized by hand or with grinders and centrifuged for 20 min at 2000–3000 r.p.m, after which the supernatant was removed. After packaging, one aliquot was tested, and the rest was frozen for later use. Standard and test sample wells were established, and 50 µl of the standard was added to the standard wells. Blank wells were also established for comparison. Neither the sample nor the HRP-conjugated reagent was added to these wells; otherwise, these wells were subjected to the same process. In total, 10 µl of the test sample was added to 40 µl of solution in each test sample well (the final dilution was 5-fold) without touching the well wall; the sample was then gently mixed.

Next, 100 µl of HRP-conjugated reagent was added to each well except for the blank wells. The plate was sealed with a membrane and incubated for 60 min at 37 °C. The plate was then uncovered, the liquid was discarded, the plate was air dried, and wash buffer (a wash solution diluted 20-fold in distilled water) was added to each well. After 30 s, the wells were drained, and the wash step was repeated 5 times before the plate was patted dry.

Chromogen Solution A (50 µl) and Chromogen Solution B were added to each well, and the plate was protected from light for 15 min at 37 °C. The reaction was stopped by adding 50 µl of a stop solution to each well (the blue colour changed to yellow). A blank well was used to zero the analyser, and the absorbance was measured at 450 nm within 15 min of adding the stop solution. The levels of intestinal inflammatory factors were calculated by comparing the OD450 values of the samples to a standard curve.

### Cardiac function scores of infants

Cardiac function was assessed using the modified Ross scale. There were 6 score indicators: the participant’s sweating position, frequency of rapid breathing, breathing condition, respiratory rate, heart rate and liver size. Each item was scored as 0, 1 or 2 according to the severity of symptoms from mild to severe. A higher score indicated more severe HF. A total score of 0 ~ 2 indicated “no HF”; a total score of 3–6 indicated “mild HF”; a total score of 7–9 indicated “moderate HF”; and a total score of 10 to 12 indicated “severe HF [13].”

### Statistical analysis

SPSS 25.0 was used to perform the statistical analysis. Categorical variables were compared by Fisher’s exact test. Comparisons between groups of continuous variables with a normal distribution deviation were performed with the T test. Continuous variables without a normal distribution deviation were compared with the Mann‒Whitney U test. Pearson’s test was used for correlation analysis between two variables.

## Results

A total of 20 HF infants with CHD were enrolled in this study. There were 11 males and 9 females aged 3.4 ± 3.8 months with a pulmonary artery pressure of 53.5 (24–85) mmHg, an NT-BNP of 5901 (596-12256), and a modified Ross score of 7.5 (5–11). In the control group, there were 12 males and 10 females aged 3.1 ± 1.9 months and with a weight of 5.4 ± 1.4 kg (Table 1).

**Table 1:** Comparison of general data between the two groups

	Heart failure group	Control group	*P* value
Age (month)	3.4 ± 2.8	3.1 ± 1.9	0.724
Male/female	11/9	10/10	0.752
Weight (kg)	4.5 ± 2.0	5.4 ± 1.4	0.105
Pulmonary arterial pressure (mmHg)	53.5 (24–85)		
NT-proBNP	5901 (596−12,256)		
Cardiac function score	7.5 (5–11)		

A comparison of intestinal inflammatory factor levels between the two groups revealed that the levels of IL-1β, IL-4, IL-6, IL-17 A and TNF-α in the HF group were much higher than those in the control group (*p* < 0.05), whereas the level of IL-10 in the HF group was lower than that in the control group (*p* < 0.05) (Table 2).

**Table 2:** Comparison of intestinal inflammatory factors between the two groups

	Heart failure group	Control group	*P* value
IL−1ß	93.87 ± 10.11	80.72 ± 10.67	0.001
IL−4	52.06 ± 2.63	47.12 ± 6.25	0.002
IL−5	91.85 ± 9.99	86.89 ± 15.98	0.247
IL−6	62.88 ± 5.39	55.21 ± 10.93	0.008
IL−8	215.83 ± 14.84	201.21 ± 34.05	0.086
IL−10	767.68 ± 84.51	823.54 ± 67.44	0.026
IL−12	37.26 ± 3.88	36.04 ± 7.69	0.530
IL−17 A	31.64 ± 3.11	28.85 ± 4.40	0.026
IFN-α	44.72 ± 4.38	44.41 ± 4.68	0.685
IFN-γ	973.87 ± 116.27	941.25 ± 212.03	0.557
TNF-α	79.01 ± 6.41	72.91 ± 10.67	0.034

**Fig. 1:**
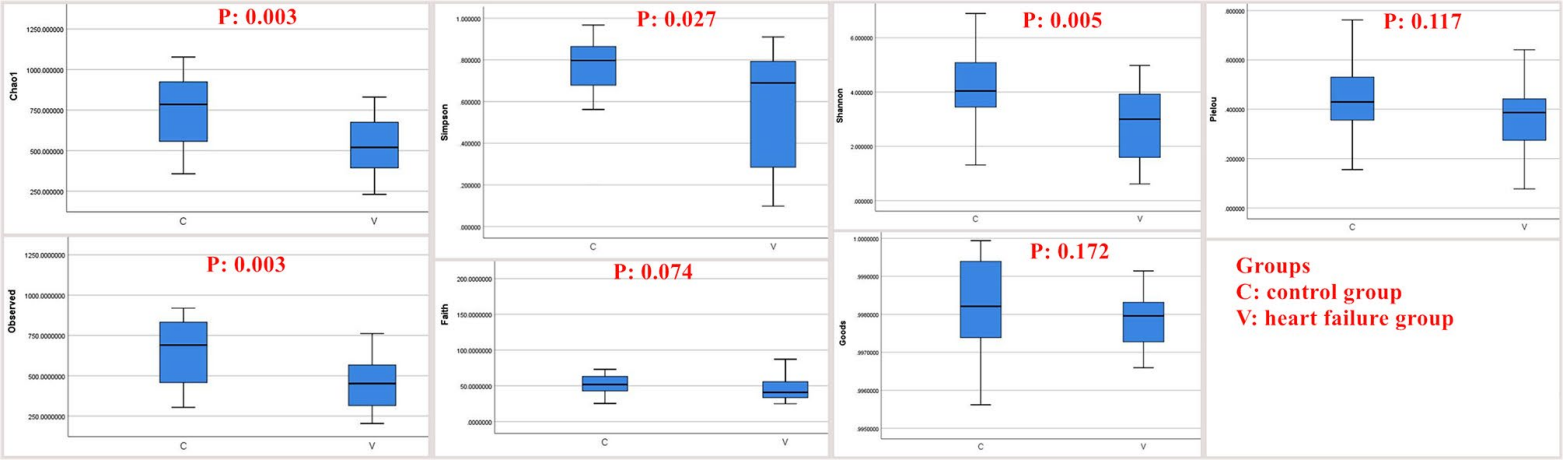
The intestinal diversity of patients in the heart failure group was significantly lower than that of patients in the control group

**Fig. 2:**
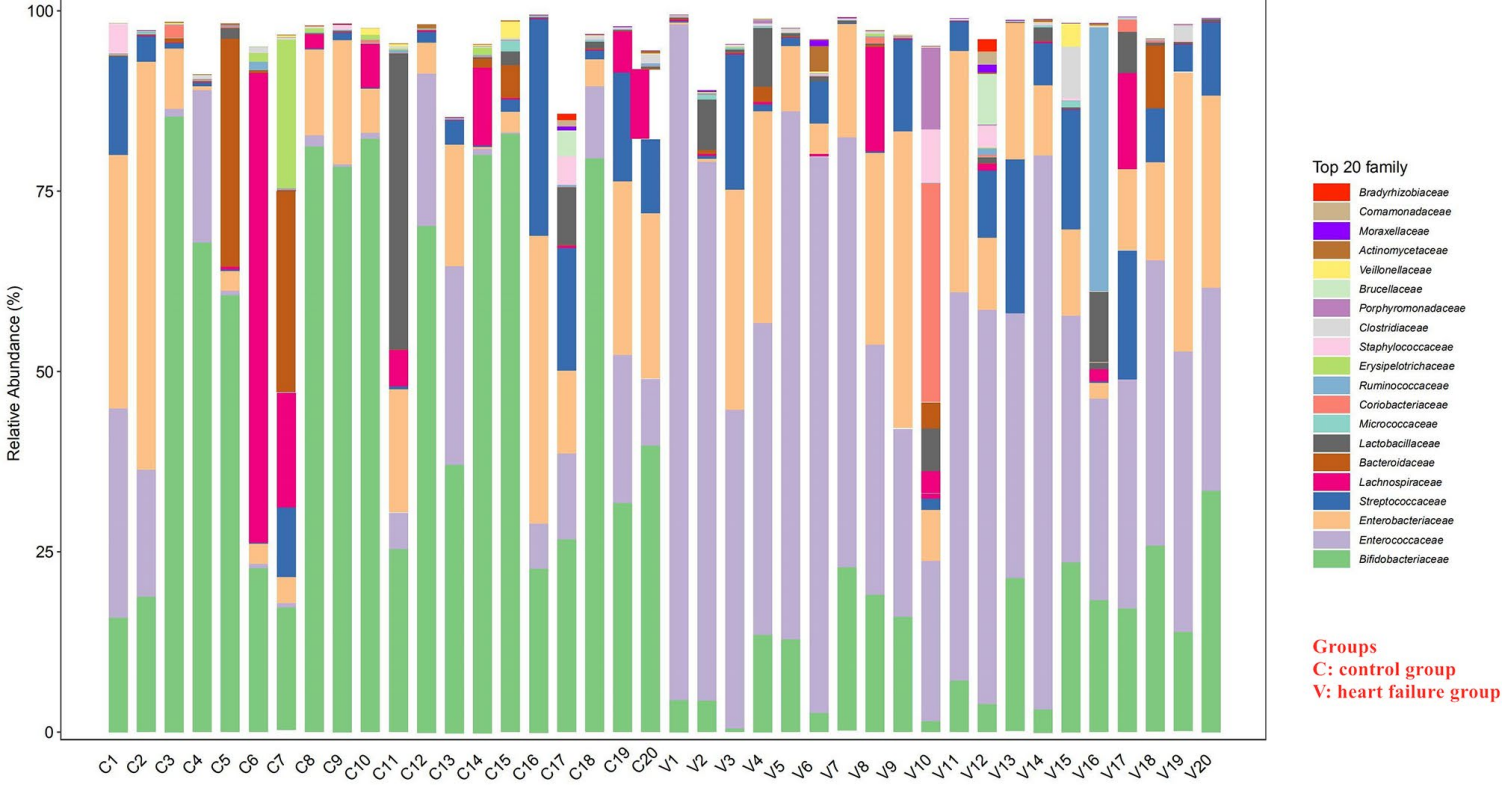
The abundance of *Enterococcus* was significantly increased in the heart failure group compared to the control group, but the abundance of *Bifidobacterium* was significantly decreased in the heart failure group compared to the control group

The intestinal microbiota data suggested that the intestinal diversity of patients in the HF group was lower than that of patients in the control group (*p* < 0.05) (Fig. 1). The abundance of *Enterococcus* was greatly increased, while that of *Bifidobacterium* was significantly decreased in the HF group compared to the control group (*p* < 0.05) (Fig. 2).

**Fig. 3:**
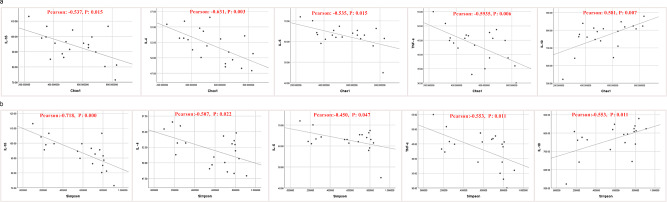
**a, b** The diversity of the intestinal microbiota was negatively correlated with the levels of IL-1β, IL-4, IL-6 and TNF-α in the intestinal tract but was positively correlated with the level of IL-10

**Fig. 4:**
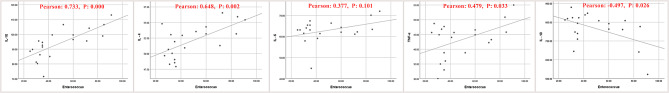
The abundance of *Enterococcus* was positively correlated with the levels of IL-1β, IL-4, IL-6 and TNF-α in the intestinal tract but was negatively correlated with the level of IL-10

The diversity of the intestinal microbiota was negatively correlated with the levels of IL-1β, IL-4, IL-6 and TNF-α in the intestinal tract but was positively correlated with the level of IL-10 (Fig. 3a and b). The abundance of *Enterococcus* was positively associated with the levels of IL-1β, IL-4, IL-6 and TNF-α in the intestinal tract but was negatively correlated with the level of IL-10 (Fig. 4).

**Fig. 5:**
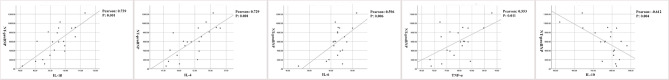
NT-proBNP was positively correlated with the levels of IL-1β, IL-4, IL-6 and TNF-α in the heart failure group but was negatively correlated with the level of IL-10

**Fig. 6:**
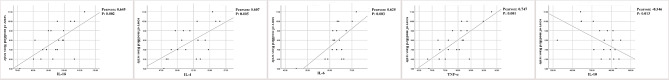
The cardiac function score was positively correlated with the levels of IL-1β, IL-4, IL-6 and TNF-α in the heart failure group but was negatively correlated with the level of IL-10

Correlation analysis between the severity of HF and the levels of intestinal inflammatory factors revealed that NT-proBNP was positively associated with the levels of IL-1β, IL-4, IL-6 and TNF-α in the HF group but was negatively correlated with the level of IL-10 (Fig. 5). The cardiac function score was positively correlated with the levels of IL-1β, IL-4, IL-6 and TNF-α in the HF group but was negatively correlated with the level of IL-10 (Fig. 6).

## Discussion

HF is characterized by reduced cardiac output and an inadequate supply of effective circulating blood, which leads to the hypoperfusion of tissues and organs [14]. The intestinal tract is the first organ to undergo ischaemia and the last organ to recover during HF [15]. Ischaemia and congestion caused by a reduced intestinal oxygen supply can result in a range of metabolic disorders and microbial disturbances. The disordered intestinal microbiota in HF patients is mainly characterized by an increase in pathogenic bacteria and a decrease in beneficial bacteria, which aggravate the intestinal inflammatory reaction and increase the levels of intestinal inflammatory factors and toxin secretion [16–18]. Our previous study revealed that heart failure in infants with congenital heart disease dysregulated the intestinal microbiota, which was characterized by an increase in pathogenic bacteria, a decrease in beneficial bacteria, and decreases in diversity and richness [19]. This study further examined the relationship between heart failure and intestinal inflammation in infants with CHD. In addition to revealing similar changes in the intestinal microbiota, this study further revealed that a decrease in the diversity of the intestinal microbiota and an increase in the abundance of *Enterococcus* were significantly correlated with an increase in intestinal inflammatory factor levels.

Numerous factors can cause the intestinal mucosal barrier to be disrupted during HF [20]. Decreased cardiac output and gastrointestinal blood hypoperfusion during HF can lead to intramucosal acidosis and intestinal wall oedema. An increase in pathogenic bacteria in the gut can damage the intestinal mucosal barrier. An increase in inflammation and toxins in the gut can also disrupt the intestinal barrier. Damaging the intestinal mucosal barrier increases intestinal permeability, and intestinal inflammatory factors enter the blood circulation through the intestinal barrier, which triggers or exacerbates the systemic inflammatory reaction and exacerbates the progression of HF [21]. This can create a vicious cycle.

This study was the first to examine the relationship between HF and intestinal inflammation in infants with CHD. Our study revealed that the levels of the intestinal inflammatory factors IL-1β, IL-4, IL-6, IL-17 A and TNF-α were markedly increased in the HF group, while the level of IL-10 was significantly decreased in the HF group. Inflammatory factors determine the pathogenesis of HF. Studies have shown that proinflammatory factors, including IL-1β, IL-4, IL-6, IL-17 A and TNF-α, have harmful effects on the heart [22, 23]. Inflammatory factors such as IL-1β, IL-6 and TNF-α downregulate the expression of Ca^2+^-regulating genes, thereby changing intracellular Ca^2+^ homeostasis, which can cause a negative inotropic effect, ultimately resulting in overt HF [24, 25]. Moreover, TNF-α, IL-1β and IL-6 can directly promote cardiomyocyte hypertrophy [26]. Increasing evidence suggests that TNF-α and IL-1β can affect cardiac function by inducing cardiomyocyte apoptosis [27, 28]. Studies have shown that IL-10 can inhibit the production of the proinflammatory factor TNF-α and prevent TNF-α-induced apoptosis [29]. Our study showed that the intestinal proinflammatory factors IL-1β, IL-4, IL-6, IL-17 A and TNF-α were robustly increased in infants with HF and CHD, while the anti-inflammatory factor IL-10 was significantly decreased. Therefore, we hypothesize that the intestinal barrier is damaged after HF and that the amount of intestinal proinflammatory factors entering the circulation is increased, which exacerbates the systemic inflammatory response and the progression of HF.

Correlation analysis between the level of intestinal inflammatory factors and the severity of HF showed that the levels of IL-1β, IL-4, IL-6 and TNF-α were positively correlated with NT-proBNP and the cardiac function score, which indicated that the increase in intestinal proinflammatory factors promoted the progression of HF and aggravated the severity of HF. We hypothesize that when HF occurs, intestinal inflammatory factor levels increase, and the intestinal barrier breaks down; subsequently, proinflammatory factors enter the circulation through the intestine, which may be associated with the progression of HF.

### Limitations

The results observed in this study might have been affected by the age of the patients, the type of HF, the single-centre nature of the study, the sample size, and the different feeding methods used. Overall, these factors demand further studies to assess their influence on the results, and we will further clarify and avoid the influence of these factors in future studies.

## Conclusions

Infants with CHD-related HF had a disordered intestinal microbiota, decreased diversity of the intestinal microbiota, increased abundance of pathogenic bacteria and decreased abundance of beneficial bacteria. An increase in the abundance of *Enterococcus* and a decrease in the diversity of the intestinal microbiota significantly aggravated the intestinal inflammatory response, which may be associated with the progression of HF. Reducing the systemic inflammatory response by reducing intestinal inflammation may be a new direction in the treatment of HF.

## Data Availability

The datasets presented in this study are deposited in online repositories. The names of the repositories and accession numbers can be found at https://www.ncbi.nlm.nih.gov/bioproject/? term = PRJNA862324.

## Abbreviations

CHD: Congenital heart disease
HF: Heart failure

## References

[1] Roger VL (2013). Epidemiology of heart failure. Circ Res.

[2] Mudd JO, Kass DA (2008). Tackling heart failure in the twenty-first century. Nature.

[3] Mosterd A, Hoes AW (2007). Clinical epidemiology of heart failure. Heart.

[4] Chioncel O, Mebazaa A, Harjola VP, Coats AJ, Piepoli MF, Crespo-Leiro MG, Laroche C, Seferovic PM, Anker SD, Ferrari R, Ruschitzka F, Lopez-Fernandez S, Miani D, Filippatos G, Maggioni AP (2017). ESC Heart failure Long-Term Registry investigators. Clinical phenotypes and outcome of patients hospitalized for acute heart failure: the ESC Heart failure Long-Term Registry. Eur J Heart Fail.

[5] von Lueder TG, Sangaralingham SJ, Wang BH, Kompa AR, Atar D, Burnett JC, Krum H (2013). Renin-angiotensin blockade combined with natriuretic peptide system augmentation: novel therapeutic concepts to combat heart failure. Circ Heart Fail.

[6] Westermann D, Lindner D, Kasner M, Zietsch C, Savvatis K, Escher F, von Schlippenbach J, Skurk C, Steendijk P, Riad A, Poller W, Schultheiss HP, Tschöpe C (2011). Cardiac inflammation contributes to changes in the extracellular matrix in patients with heart failure and normal ejection fraction. Circ Heart Fail.

[7] Libby P, Nahrendorf M, Swirski FK (2016). Leukocytes Link Local and systemic inflammation in Ischemic Cardiovascular Disease: an expanded Cardiovascular Continuum. J Am Coll Cardiol.

[8] Frangogiannis NG (2014). The inflammatory response in myocardial injury, repair, and remodelling. Nat Rev Cardiol.

[9] van den Hoogen P, van den Akker F, Deddens JC, Sluijter JP (2015). Heart failure in chronic myocarditis: a role for microRNAs?. Curr Genomics.

[10] Sender R, Fuchs S, Milo R (2016). Are we really vastly outnumbered? Revisiting the ratio of bacterial to Host Cells in humans. Cell.

[11] Nicholson JK, Holmes E, Kinross J, Burcelin R, Gibson G, Jia W, Pettersson S (2012). Host-gut microbiota metabolic interactions. Science.

[12] Hinton RB, Ware SM (2017). Heart failure in Pediatric patients with congenital heart disease. Circ Res.

[13] Läer S, Mir TS, Behn F, Eiselt M, Scholz H, Venzke A, Meibohm B, Weil J (2002). Carvedilol therapy in pediatric patients with congestive heart failure: a study investigating clinical and pharmacokinetic parameters. Am Heart J.

[14] Jia Q, Xie Y, Lu C, Zhang A, Lu Y, Lv S, Zhang J (2019). Endocrine organs of cardiovascular diseases: gut microbiota. J Cell Mol Med.

[15] Takala J (1996). Determinants of splanchnic blood flow. Br J Anaesth.

[16] Salomon J, Ericsson A, Price A, Manithody C, Murry DJ, Chhonker YS, Buchanan P, Lindsey ML, Singh AB, Jain AK (2021). Dysbiosis and Intestinal Barrier Dysfunction in Pediatric congenital heart disease is exacerbated following cardiopulmonary bypass. JACC Basic Transl Sci.

[17] Mann DL (2015). Innate immunity and the failing heart: the cytokine hypothesis revisited. Circ Res.

[18] Kummen M, Mayerhofer CCK, Vestad B, Broch K, Awoyemi A, Storm-Larsen C, Ueland T, Yndestad A, Hov JR, Trøseid M (2018). Gut microbiota signature in heart failure defined from profiling of 2 independent cohorts. J Am Coll Cardiol.

[19] Zhang QL, Chen XH, Zhou SJ, Lei YQ, Huang JS, Chen Q, Cao H (2023). Relationship between disorders of the intestinal microbiota and heart failure in infants with congenital heart disease. Front Cell Infect Microbiol.

[20] Lewis CV, Taylor WR (2020). Intestinal barrier dysfunction as a therapeutic target for cardiovascular disease. Am J Physiol Heart Circ Physiol.

[21] Nagatomo Y, Tang WH (2015). Intersections between Microbiome and Heart failure: revisiting the gut hypothesis. J Card Fail.

[22] Seta Y, Shan K, Bozkurt B, Oral H, Mann DL (1996). Basic mechanisms in heart failure: the cytokine hypothesis. J Card Fail.

[23] Deswal A, Petersen NJ, Feldman AM, Young JB, White BG, Mann DL (2001). Cytokines and cytokine receptors in advanced heart failure: an analysis of the cytokine database from the Vesnarinone trial (VEST). Circulation.

[24] Wu CK, Lee JK, Chiang FT, Yang CH, Huang SW, Hwang JJ, Lin JL, Tseng CD, Chen JJ, Tsai CT (2011). Plasma levels of tumor necrosis factor-α and interleukin-6 are associated with diastolic heart failure through downregulation of sarcoplasmic reticulum Ca2 + ATPase. Crit Care Med.

[25] Sedej S, Schmidt A, Denegri M, Walther S, Matovina M, Arnstein G, Gutschi EM, Windhager I, Ljubojević S, Negri S, Heinzel FR, Bisping E, Vos MA, Napolitano C, Priori SG, Kockskämper J, Pieske B (2014). Subclinical abnormalities in sarcoplasmic reticulum ca(2+) release promote eccentric myocardial remodeling and pump failure death in response to pressure overload. J Am Coll Cardiol.

[26] Savvatis K, Müller I, Fröhlich M, Pappritz K, Zietsch C, Hamdani N, Grote K, Schieffer B, Klingel K, Van Linthout S, Linke WA, Schultheiss HP, Tschöpe C (2014). Interleukin-6 receptor inhibition modulates the immune reaction and restores titin phosphorylation in experimental myocarditis. Basic Res Cardiol.

[27] Condorelli G, Morisco C, Latronico MV, Claudio PP, Dent P, Tsichlis P, Condorelli G, Frati G, Drusco A, Croce CM, Napoli C (2002). TNF-alpha signal transduction in rat neonatal cardiac myocytes: definition of pathways generating from the TNF-alpha receptor. FASEB J.

[28] Takahashi M (2014). NLRP3 inflammasome as a novel player in myocardial infarction. Int Heart J.

[29] Dhingra S, Bagchi AK, Ludke AL, Sharma AK, Singal PK (2011). Akt regulates IL-10 mediated suppression of TNFα-induced cardiomyocyte apoptosis by upregulating Stat3 phosphorylation. PLoS ONE.

